# Correction: E2SVM: Electricity-Efficient SLA-aware Virtual Machine Consolidation approach in cloud data centers

**DOI:** 10.1371/journal.pone.0346744

**Published:** 2026-04-08

**Authors:** Vaneet Kumar, Aleem Ali, Payal Mittal, Ibrahim Aqeel, Mohammed Shuaib, Shadab Alam, Mohammed Y. Aalsalem

The articles cited as References 7, 9 and 28 were erroneously included and should not be cited in the article [1], and Reference 10 is not appropriate.

The replacement references for 7, 9, 10 and 28 are cited here as [2–5], respectively.

The authors apologize for the errors in the published article [1].
