# Supplementary material for: E2SVM: Electricity-Efficient SLA-aware Virtual Machine Consolidation approach in cloud data centers
Source: PLoS One. 2024 Jun 10;19(6):e0303313. doi: 10.1371/journal.pone.0303313 (PMC11164395; doi:10.1371/journal.pone.0303313)
Supplement: Attachment — Submitted filename: Reply to Reviewer comments E2VSM.docx [file pone.0303313.s001.docx]

**Response to Review Comments**

**Title:** E2SVM: Electricity-Efficient SLA-aware Virtual Machine Consolidation Approach in Cloud Data Centers

**Journal:** Plos One

**Article ID:** PONE-D-24-01820

We are thankful to the editor and reviewers for the careful and thorough reading of this paper as well as for constructive suggestions. This helps us to improve the quality of our paper and future publications.

**Reviewer #1**

| **S. No.** | **Review Comments** | **Response to reviewer comments** |
| --- | --- | --- |
| 1. | The Abstract of this paper should be rewritten. | The abstract has been updated and highlighted. |
| 2. | In the paper, what is the time complexity for the proposed algorithm? It is better for the authors to give more explanations of the algorithms, such as the function of each step, and the overall basic idea. | As per the suggestion of the reviewer, the abstract has been revised and changes are highlighted in the revised manuscript |
| 3. | The authors make a comparison with other algorithms in the paper. However, these algorithms are out of date. It is not fair to make a comparison with these algorithms. Could the authors make a comparison with the newly published algorithms in the field of VM deployment | As advised, comparisons are made with the latest MuMs approach published in 2017. |
| 4. | For the comparison algorithms, It is suggested to make a simple introduction in the experiment part. | As suggested a brief introduction of baseline approaches is added and highlighted in the results and discussion section. |
| 5. | The quality of the Figures in the paper should be further improved in the revision. | As per the recommendation, the high-quality Figures have been added. |
| 6. | In the paper, the authors evaluate the energy consumption. However, any power model is essential for energy-aware algorithms. Have the authors considered selecting other energy consumption models? | As suggested power model is added in the experiment setup section and highlighted. |
| 7. | There are many spelling errors in the manuscript. A thorough spelling check is required in the revision. | The manuscript is thoroughly checked and corrected all spelling errors in the improved version. |

**Reviewer #2**

| **S. No.** | **Review Comments** | **Response to reviewer comments** |
| --- | --- | --- |
| 1. | Given that energy consumption is a central topic of the paper, I think the energy model should be motivated and explained better to convince the reader that energy consumption is linearly proportional to CPU utilisation. The cited energy model refers to a study from 2007 and I believe that data center technologies are changed in the meantime. Moreover, my intuition is that such linear relation is referred to CPU-bound applications only, and I do not know if can be considered for the general case. I suggest studying and discussing these points in the paper. Moreover, I think that the used algorithm's execution should be evaluated in terms of energy consumption since the result of about 9% energy consumption in the evaluation section could be decreased by the algorithms. E.g. if the algorithms execution consumes 10% of the overall energy there is no gain in terms of energy consumption. | As suggested energy consumption model is added in the experiment setup section and highlighted. The energy consumption CPU utilization equation is also written and cited. |

**Reviewer #3**

| **S. No.** | **Review Comments** | **Response to reviewer comments** |
| --- | --- | --- |
| 1. | Although the ABSTRACT structure is good, I suggest that the philosophy of using the proposed method should be explained. | ABSTRACT is updated with the philosophy of using the proposed method. |
| 2. | In my opinion, the INTRODUCTION section needs to be revised. In this section there should be three points: 1) motivation, 2) a summary of the challenges of previous studies, and 3) contribution. | The INTRODUCTION section is rewritten and add more points as suggested. The updates are highlighted. |
| 3. | The Related Work section is too short, should have additional resources, and may be separated by subjects: QoS, Energy consumption, and SLA, subjects related to the article. | Thanks for providing the latest work relevant to our study, the related papers are studied and mentioned in the related work section and highlighted. |
| 4. | it is better to analyze the time complexity of the proposed method in the worst case. | The time complexity theorem of proposed method is analyzed and added at the end of the material and method section. Added context is highlighted. |
| 5. | The main limitation of the proposed methods should be explained in detail; | The main limitation of the proposed method is explained in the last paragraph of related work and highlighted. |
| 6. | The contribution and novelty of this work should be further justified and future studies should be given; | The main contribution and novelty of the proposed method are explained with the help of an architectural diagram at the start of the materials and method section. |
| 7. | The simulation environment should be provided in detail, e.g., software, sampling size, solver type, etc. | The simulation environmental setup is drafted in part a of the experimental setup section. Software, sampling size is mentioned in the first paragraph of the result and discussion section and highlighted. |
| 8. | An important point needs to be included in this article: the type of application that can be executed using the proposed model. The inclusion of this facet would enrich the article's scope and applicability; | The type of application is mentioned in the experimental setup section. |
| 9. | There are still some grammatical errors in the manuscript. Authors should use software such as Grammarly for proof-checking. | After proofreading, all the Typo and grammatical errors are corrected. |
| 10. | The tense of the verbs in the CONCLUSION section must be past tense. In this section, the most important numerical improvements of the proposed method should be mentioned and marginal explanations should be avoided. In addition,  the suggestions mentioned for further research should be well presented. | As per suggestions the conclusion is rewritten, and changes are highlighted. |
